# Capturing and Operationalizing Participation in Pediatric Re/Habilitation Research Using Artificial Intelligence: A Scoping Review

**DOI:** 10.3389/fresc.2022.855240

**Published:** 2022-04-14

**Authors:** Vera C. Kaelin, Mina Valizadeh, Zurisadai Salgado, Julia G. Sim, Dana Anaby, Andrew D. Boyd, Natalie Parde, Mary A. Khetani

**Affiliations:** ^1^Rehabilitation Sciences, University of Illinois at Chicago, Chicago, IL, United States; ^2^Children's Participation in Environment Research Lab, University of Illinois at Chicago, Chicago, IL, United States; ^3^Computer Science, University of Illinois at Chicago, Chicago, IL, United States; ^4^Natural Language Processing Laboratory, University of Illinois at Chicago, Chicago, IL, United States; ^5^School of Physical and Occupational Therapy, McGill University, Montreal, QC, Canada; ^6^CanChild Centre for Childhood Disability Research, McMaster University, Hamilton, ON, Canada; ^7^Biomedical and Health Information Sciences, University of Illinois at Chicago, Chicago, IL, United States; ^8^Physical Therapy, University of Illinois at Chicago, Chicago, IL, United States; ^9^Occupational Therapy, University of Illinois at Chicago, Chicago, IL, United States

**Keywords:** involvement, engagement, assessment, measurement, natural language processing, machine learning, computer vision, technology

## Abstract

**Background:**

There is increased interest in using artificial intelligence (AI) to provide participation-focused pediatric re/habilitation. Existing reviews on the use of AI in participation-focused pediatric re/habilitation focus on interventions and do not screen articles based on their definition of participation. AI-based assessments may help reduce provider burden and can support operationalization of the construct under investigation. To extend knowledge of the landscape on AI use in participation-focused pediatric re/habilitation, a scoping review on AI-based participation-focused assessments is needed.

**Objective:**

To understand how the construct of participation is captured and operationalized in pediatric re/habilitation using AI.

**Methods:**

We conducted a scoping review of literature published in Pubmed, PsycInfo, ERIC, CINAHL, IEEE Xplore, ACM Digital Library, ProQuest Dissertation and Theses, ACL Anthology, AAAI Digital Library, and Google Scholar. Documents were screened by 2–3 independent researchers following a systematic procedure and using the following inclusion criteria: (1) focuses on capturing participation using AI; (2) includes data on children and/or youth with a congenital or acquired disability; and (3) published in English. Data from included studies were extracted [e.g., demographics, type(s) of AI used], summarized, and sorted into categories of participation-related constructs.

**Results:**

Twenty one out of 3,406 documents were included. Included assessment approaches mainly captured participation through annotated observations (*n* = 20; 95%), were administered in person (*n* = 17; 81%), and applied machine learning (*n* = 20; 95%) and computer vision (*n* = 13; 62%). None integrated the child or youth perspective and only one included the caregiver perspective. All assessment approaches captured behavioral involvement, and none captured emotional or cognitive involvement or attendance. Additionally, 24% (*n* = 5) of the assessment approaches captured participation-related constructs like activity competencies and 57% (*n* = 12) captured aspects not included in contemporary frameworks of participation.

**Conclusions:**

Main gaps for future research include lack of: (1) research reporting on common demographic factors and including samples representing the population of children and youth with a congenital or acquired disability; (2) AI-based participation assessment approaches integrating the child or youth perspective; (3) remotely administered AI-based assessment approaches capturing both child or youth attendance and involvement; and (4) AI-based assessment approaches aligning with contemporary definitions of participation.

## Introduction

Participation is a key re/habilitation outcome that has been defined by the World Health Organization as the “involvement in life situation” (1, p. 9). In pediatric re/habilitation this definition has been further conceptualized by Imms et al. (2) in the family of Participation-Related Constructs (fPRC) framework. This contemporary framework defines participation as child or youth attendance and involvement in activities, which is related to but distinct from their activity competencies, environment/context, and preferences or sense of self (2, 3). Attendance is the objective dimension of participation and has been commonly used to quantify participation in pediatric re/habilitation (2–5). Involvement is considered as more complex (2, 3, 5) and has been further grouped by education and pediatric re/habilitation literature into behavioral, cognitive, and emotional involvement (6–8). Behavioral involvement is considered observable on-task behavior (8), whereas cognitive involvement (thoughtfulness and willingness to employ effort for tasks) and emotional involvement (positive and negative feelings when interacting with people or tasks) are non-observable (3, 8).

Recent literature reviews revealed inconsistent conceptualization of participation in pediatric re/habilitation, hindering interpretability and comparison across studies and practice approaches (4, 5). For example, participation has often been used interchangeably with activity competence, rendering confusion about these two distinct but related constructs (2–5). For efficient service provision (9) to reduce costs and provider and patient burden (10), there is need to simplify processes within participation-focused pediatric re/habilitation services without compromising the complexity and the customization of participation-focused services to individual needs.

The application of artificial intelligence (AI), which is considered a top re/habilitation research priority (9, 11), might be one way to address this need. AI can be defined as systems that think and act rationally by mimicking humans (12). Regardless of the type of AI method employed [e.g., machine learning (ML), natural language processing (NLP)] (12), AI is commonly used to simplify processes and to customize information to individuals' preferences and needs, which could benefit the healthcare industry (13). In pediatric re/habilitation, AI may help to consolidate and analyze information in ways that afford for providers to more efficiently enact the evaluation and goal-setting, intervention, and reevaluation phases of the therapeutic process (14) to deliver client-centered and participation-focused re/habilitation interventions (15, 16). In the last decade there has been a vast increase in research on the use of AI in participation-focused pediatric re/habilitation warranting need for summarizing the body of literature in this area of work (17).

Recently, our scoping review (17) on the use of AI in re/habilitation interventions targeting the participation of children and youth with acquired and congenital disabilities included appraisal of: (1) their type of AI and customization used; (2) their mode of delivery (i.e. in-person, remote); and (3) whether goal-setting was addressed. Results revealed 94 studies using AI in participation-focused pediatric re/habilitation interventions. Of these 94 studies, only 7 (8%) applied types of AI other than robotics or virtual reality (VR), only one study (1%) was tailored to patients' individual needs, only 10 (11%) were delivered remotely, and only one (1%) of the studies described individual goal-setting as part of their intervention (17).

A main limitation of this scoping review include its exclusive focus on interventions (17). Assessments that are conceptually sound play a substantial role in shaping the enactment of quality therapeutic processes (4, 5, 14, 18) and ensuring consistent interpretation of research findings across studies (5). Prior systematic reviews revealed few participation assessments that aligned with contemporary definitions of child and youth participation [i.e., children and youth's attendance and their involvement (2, 3)] (5, 19). Despite increased interest in using AI to capture participation (15), none of the included pediatric assessment approaches used AI (5, 19). The lack of AI to assess for child and youth participation might be due to selection of search terms and differing terminology in pediatric re/habilitation and computer science (e.g., while “measure” is used for clinical assessment approach in pediatric re/habilitation, it is often a data analytic term in computer science). Alternatively, the use of AI to assess children's participation is still in a nascent phase, which could have precluded their inclusion. Additionally, the authors did not examine participation assessments in regards to their focus on types of involvement (i.e., behavioral, cognitive, and emotional) and concluded the need for “further investigation and characterization, both in relation to what constitutes involvement and the best methods of measurement.” (5, p. 13). To extend knowledge of the landscape on AI use in participation-focused pediatric re/habilitation, a scoping review on AI-based participation-focused assessments is needed.

Therefore, the purpose of this scoping review was to understand how the construct of participation is captured and operationalized in pediatric re/habilitation using AI, and to what extent it aligns with the contemporary definitions of child and youth participation [i.e., attendance and involvement (2, 3), as indicated by child or youth behavioral, cognitive, and emotional involvement (6–8)].

## Methods

### Study Design

We conducted a scoping review to summarize the breadth of existing evidence on how participation is captured and operationalized in pediatric re/habilitation research using AI-based assessment approaches and to identify gaps for future research (20–22). In re/habilitation disciplines, assessment is a way to gather clinically relevant information about a patient (23). This can be done via different modalities (e.g., observation, interview) and through standardized or non-standardized tools. For this review, assessment is considered an approach and does not necessarily include a standardized tool. We use the PRISMA-ScR checklist (22) and the Joanna Briggs Institute guidelines by Peter et al. (21), encompassing an enhanced version of Arksey and O'Malley's five steps (21, 24, 25). A protocol for this scoping review is registered in Open Science Framework (26).

### Step 1: Identifying the Research Question(s)

How is child or youth participation captured and operationalized in participation-focused pediatric re/habilitation research using AI?

a) What are the demographic characteristics of the targeted population examined in studies using AI to capture participation?
b) What types of AI have been used to assess for child and youth participation in pediatric re/habilitation research?
c) What methods (i.e., reported, observation, estimates), data sources (i.e., child/youth, caregiver, researcher, re/habilitation professional, other type of professional/not specified, facial/skeleton/eye recognition, sensors, Electroencephalogram (EEG), distance estimate, other), and mode of administration (i.e., remotely, in person) have been used to assess for child and youth participation in pediatric re/habilitation?
d) To what extent does participation-focused pediatric re/habilitation research using AI assess for participation in ways that align with the contemporary definition of child and youth participation (2, 3, 6–8)?
e) What are the research gaps in addressing child and youth participation, as aligned with the contemporary definition of child and youth participation, in pediatric re/habilitation research that uses AI?

### Step 2: Identifying Relevant Studies

The first author of this review (VK) conducted a systematic literature search in well-established applied health sciences and computer science databases (i.e., Pubmed, PsycInfo, ERIC, CINAHL, IEEE Xplore, ACM Digital Library) with additional searches in ACL Anthology and AAAI Digital Library to retrieve documents published before February 2021. No search limitations were applied, including no publication data limit. We used a search strategy previously published by Kaelin et al. (17). For this scoping review, we additionally conducted a search for gray literature in Google Scholar (200 most relevant) (27) and ProQuest Dissertation and Theses, and we screened the reference lists of included studies (see Appendix 1 for exemplar search history for gray literature search).

### Step 3: Study Selection

Documents were included if: (1) the document included a focus on capturing participation using AI; (2) the research paper included data on children and/or youth [aged 0–24 years, as aligned with the definition of children and youth put forth by the United Nations (28)] with a congenital or acquired disability (1); and (3) the document was published in English. No operational definition of participation was applied, so as to ensure inclusion of a broad scope of documents. The following terms have been used to describe participation in the fPRC (2, 3) and/or in prior literature reviews on pediatric participation (4, 19, 29) and were therefore considered as indicators of participation and included in this review: participation, inclusion, engagement, playfulness, access or attendance to life situation/settings/activities, social interaction, and social engagement. Documents were excluded if: (1) the document did not include a focus on capturing participation in daily activities (e.g., focus was on measuring skill development); (2) there was no use of AI to capture participation; (3) there were no data included of children or youth with a congenital or acquired disability (1); (4) the document focused on data of adults (mean age >24 years) (28); (5) the document was published in languages other than English; or (6) the document was a textbook review, textbook chapter, literature review, study protocol or demonstration paper, conference or workshop program, or included only an abstract without additional information. To prevent missing relevant documents, the reference lists of excluded literature reviews were screened.

**Figure 1:**
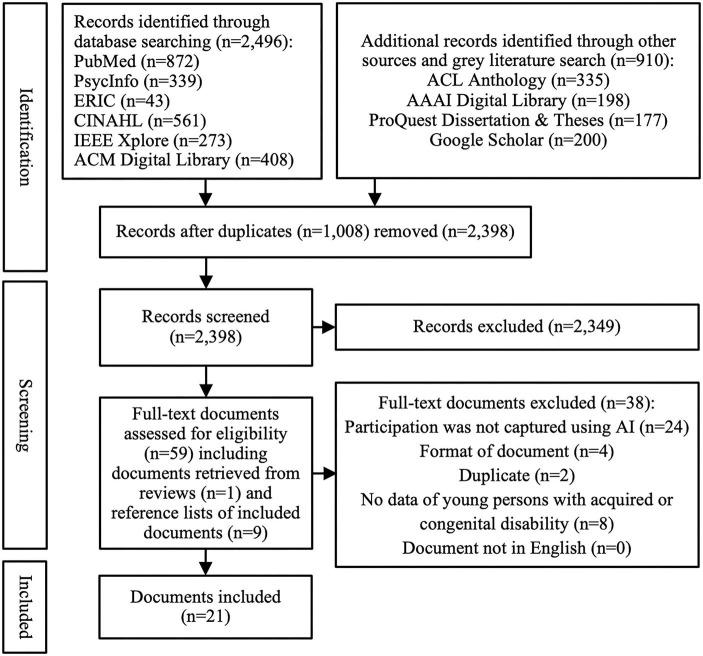
Study selection.

After removal of duplicates from the scientific and gray literature search (*n* = 1,008), the titles and abstracts of 2,398 documents were screened for inclusion by two researchers independently (VK and MV) (see Figure 1). This resulted in 49 documents that underwent full-text screening by three researchers independently (ZS, JS, and VK) based on the same inclusion and exclusion criteria as for title and abstract screening. Disagreements during title/abstract and full-text screening were resolved through discussion and key informant feedback (MK and NP). In addition, a total of 86 documents were identified through title screening of reference lists in both included documents and excluded literature reviews. After abstract screening of these 86 additional documents, 10 were identified for full-text screening based on the same inclusion and exclusion criteria.

### Step 4: Charting the Data

For all included documents, data were extracted by the same three researchers using Microsoft Excel, based on the following categories: Author(s), year, title, sample size, child/youth age, child/youth gender, child/youth acquired or congenital disability, child/youth race and ethnicity [Hispanic, non-Hispanic], family socio-economic status, family income, parental education level, how participation is operationalized, term(s) used to denote participation, whether a definition was provided for participation, participation activity addressed, approach to data collection (i.e., reported, observation, estimates), data source(s) (i.e., child/youth, caregiver, researcher, re/habilitation professional, other type of professional/not specified, facial/skeleton/eye recognition, sensors, EEG, distance estimate, other), type(s) of AI used [i.e., cognitive modeling, computer vision, constraint satisfaction and optimization, game theory, human-agent/computer/robot interaction, human computation and crowdsourcing, knowledge representation and reasoning, ML, NLP, planning/routing/scheduling, robotics, and visualization and VR (12)], and mode of administration (i.e., remotely, in person). The selection of demographic categories for extraction was guided by prior research on common predictors of child and youth participation (30–32). To ensure clarity and relevance of these categories, the data extraction tool was first trialed by three researchers (VK, ZS, and JS) with 5 included documents selected at random.

### Step 5: Collating, Summarizing, and Reporting Results

**Figure 2:**
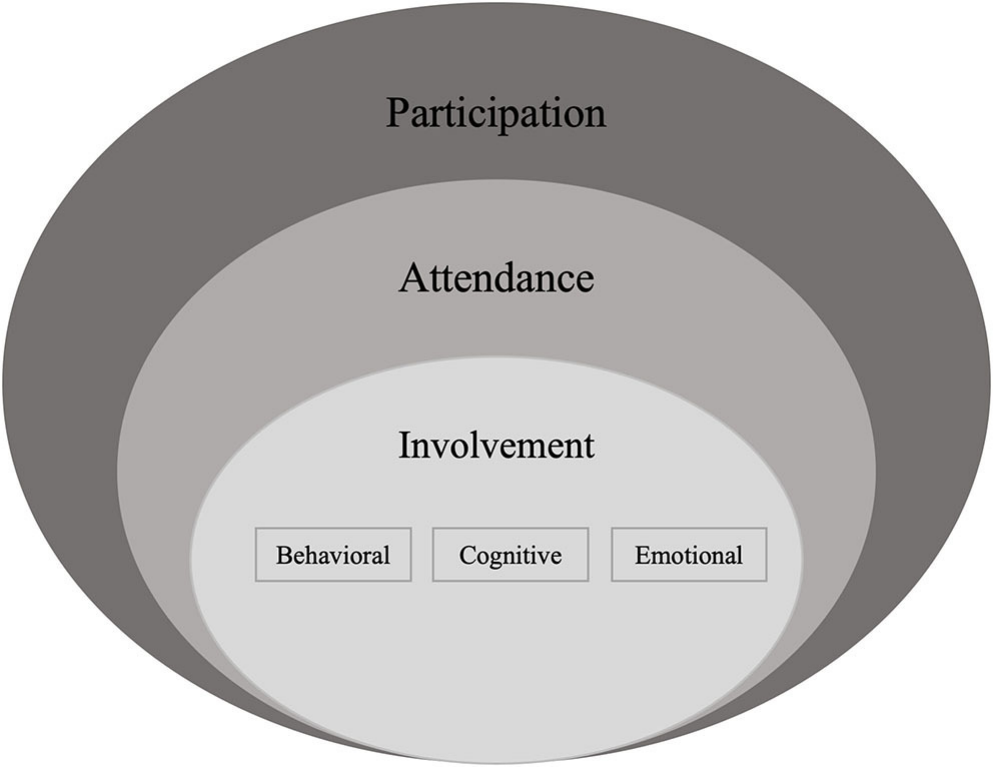
Conceptualization of participation based on contemporary frameworks of participation. Informed by the family of Participation-Related Constructs (fPRC) (2, 3) as paired with research on the conceptualization of involvement (6–8).

Following data charting, we summarized the included studies according to their publication date, sample size, included child and youth age, gender, acquired or congenital disability, race and ethnicity, and their family's socio-economic status and/or income and their parents' education level. We calculated frequencies for the approach to data collection, data source(s), the type(s) of AI used, mode of administration, and whether a definition for participation was provided. Additionally, the first author (VK) sorted the data in the category of how participation is operationalized according to the fPRC (2, 3) paired with research on the conceptualization of involvement (6–8) and as visualized in Figure 2. More specifically, data was mapped to (1) child and youth attendance, (2) their involvement (2, 3), as indicated by child or youth behavioral, cognitive, and emotional involvement (6–8), (3) participation-related constructs (i.e., activity competence, sense of self, preferences, environment/context), and 4) a category “other” in situations where data could neither be mapped to participation (i.e., attendance, type of involvement) nor participation-related constructs. For participation assessment approaches that focused on involvement without specifying the type, we assumed a focus on all types of involvement. Uncertainties were discussed with a key informant (MK).

## Results

Our scientific and gray literature search revealed 3,406 documents, with 1,008 duplicates, resulting in 2,398 documents that we screened based on their title and abstract (see Figure 1). A total of 2,349 documents were excluded, resulting in 49 documents that underwent full-text screening, and another 10 documents that were identified by screening the reference lists of excluded literature reviews and included studies. While most documents were excluded because they did not use AI to capture participation (*n* = 24), additional reasons for exclusion were the lack of data on children and/or youth with a congenital or acquired disability (*n* = 8), document format (i.e., protocol or demonstration papers, only an abstract was available) (*n* = 4), and duplicates (e.g., a study from a dissertation already included in form of a published article) (*n* = 2). This resulted in 21 included studies for this scoping review, each representing a different AI-based participation assessment approach.

### Demographic Characteristics of the Included Samples

We describe the included studies based on their publication date, included sample size, sampled child and/or youth age, gender, congenital or acquired disability, and race and/or ethnicity [Hispanic, non-Hispanic], as well as the family's socio-economic status or income and the parental education level.

The 21 included studies were published between 2007 and 2020, with most of them (*n* = 13/21; 62%) published after 2016 (33–45). The sample size of the included studies ranged from 2 to 35 children and/or youth, with a mean age up to 20.8 years (see Table 1). Of the 12 studies that reported on child and/or youth gender, 11 studies (92%) included male majority samples (33, 34, 38–41, 45, 46, 49, 50, 53). The vast majority of the included studies focused on children and/or youth with autism spectrum disorder (ASD) (*n* = 19/21; 91%) (33, 34, 37–53), followed by single instances of studies including children with Down syndrome (5%) (50), children with a visual impairment (5%) (35), and children with cerebral palsy (5%) (36). None of the studies reported on family socio-economic status, family income, or child or youth ethnicity [Hispanic, non-Hispanic]. Only 1 study reported on child or youth race (33) and only 1 study reported on parental education level for one of the caregivers sampled (40).

**Table 1:** Included studies.

**References**	**Sample (*n*)**	**Child/youth age, Mean(SD); range [years]**	**Child/youth** **gender, male**	**Child/youth** **diagnosis**	**Race/ethnicity, socio-** **economic status, parental** **education, or family income**
Ahmed et al. (33)	7 children	M (SD) = 12.7; range = 8–19	57%	ASD	71% White, 29% AA
Bian (34)	30 youth	M (SD) = 15.2	93%	ASD	NR
Chorianopoulou et al. (38)	17 children	Range = 1.2–6.7	82%	ASD	NR
Fan et al. (46)	16 youth	M (SD) = 15.2 (1.6); range = 13–18	100%	ASD	NR
Fan et al. (39)	20 youth	M (SD) = 15.3 (1.7)	95%	ASD	NR
Feil-Seifer et al. (47)	8 children	NR	NR	ASD	NR
Feil-Seifer et al. (48)	13 children	NR	NR	ASD	NR
Feil-Seifer et al. (49)	8 children	Children: range = 5–10; youth: M (SD) = 20.8	Children: NR	ASD, TD	NR
	and 7 youth		Youth: 86%
Feng et al. (40)	2 children	M (SD) = 4.5 (0.7); range = 4–5	100%	ASD	Greater than high school
Fleury (36)	5 children	M (SD) = 3.8 (1.8); range = 2–6	20%	CP, TD	NR
Ge et al. (50)	3 children	M (SD) = 12.3 (1.5); range = 11–14	100%	ASD, DS	NR
Hashemi et al. (41)	33 children	M (SD) = 2.2	88%	ASD, TD	NR
Kalantarian et al. (42)	13 children	M (SD) = 6.9 (2.5)	NR	ASD	NR
Khamassi et al. (43)	12 children	NR	NR	ASD	NR
Krupa et al. (51)	20 children	NR	NR	ASD	NR
Lahiri et al. (52)	8 youth	M (SD) = 16.1 (2.1); range = 13–18.3	NR	ASD	NR
Liu et al. (53)	3 youth	M (SD) = 14.3 (1.2); range = 13–15	100%	ASD	NR
Rudovic et al. (37)	30 children	Range = 3–13	NR	ASD	NR
Rudovic et al. (44)	35 children	Range = 3–13	NR	ASD	NR
Rudovic et al. (45)	35 children	M (SD) = 8.5; range = 3–13	82%	ASD	NR
Volta et al. (35)	17 children	NR	NR	VI	NR

*AA, African American; DS, Down syndrome; ASD, autism spectrum disorder; CP, cerebral palsy; VI, visual impairment; NR, not reported*.

### Capturing Participation

We synthesize findings about capturing participation, according to how existing AI-based assessment approaches gathered data, the data source(s) used, and the type(s) of AI used to capture participation.

Of the 21 assessment approaches, 20 (95%) (34–53) used annotated observations of child and youth participation as collected by the re/habilitation professional (*n* = 8/20; 40%) (34, 35, 39, 40, 43, 45, 52, 53), researcher (*n* = 5/20; 25%) (35, 36, 40, 46, 47), or other type of professional (e.g., psychologist, expert human rater) (*n* = 12/20; 60%) (34, 35, 37, 38, 40–42, 44, 48–51) to include in predictive models of participation (see Table 2). More specifically, re/habilitation professionals, researchers or other types of professionals rated the child or youth participation via observation in real-time or on video and then a classification model was trained to predict participation based on that labeled data. None of the included participation assessment approaches included the child or youth perspective (e.g., annotations conducted by the child or youth, self-reported data), and only one included the caregiver perspective (i.e., annotated observations by caregivers) in their predictive model of participation (40). Liu et al. (53) collected youth and caregiver report data (i.e., annotations done by youth and caregiver), in addition to therapist annotations; however, only therapist annotations together with collected physiological indices (e.g., heart sound) were included in the predictive model of participation.

**Table 2:** Data collection, data source, and type(s) of AI used.

**References**	**Data collection method and source**										**Type(s) of AI used to**
											**capture participation**
	**Reported**	**Observation**				**Estimates**					
				**Re/habilitation**	**Other type of professionals,**	**Facial/skeleton/**			**Distance**
	**Child/youth**	**Caregiver**	**Researcher**	**professional**	**not specified**	**eye recognition**	**Sensors**	**EEG**	**estimate**	**Other**	
Ahmed et al. (33)						X					CV
Bian (34)				X	X		X	X		X	Exp. 1: CV, ML, VR
											Exp. 2: ML, VR
Chorianopoulou et al. (38)					X					X	ML, NLP
Fan et al. (46)			X					X			ML, VR
Fan et al. (39)				X				X			ML, VR
Feil-Seifer et al. (47)			X						X		CV, ML, R, HCI
Feil-Seifer et al. (48)					X				X		CV, ML, R, HCI
Feil-Seifer et al. (49)					X				X		CV, ML, R, HCI
Feng et al. (40)		X	X	X	X	X	X			X	ML, R, HCI
Fleury (36)			X					X			ML, R, HCI
Ge et al. (50)					X	X					CV, ML
Hashemi et al. (41)					X	X					CV, ML
Kalantarian et al. (42)					X	X					CV, ML
Khamassi et al. (43)				X						X	ML, R, HCI
Krupa et al. (51)					X		X				ML
Lahiri et al. (52)				X		X					CV, ML, VR
Liu et al. (53)	X	X		X			X				ML
Rudovic et al. (37)					X	X					CV, ML, R, HCI
Rudovic et al. (44)					X	X	X			X	CV, ML, R, HCI
Rudovic et al. (45)				X		X	X			X	CV, ML, R, HCI
Volta et al. (35)			X	X	X						CV, ML
Total (*n*)	1	2	5	8	12	9	6	4	3	6	ML= 20; CV = 13; R = 9;
											HCI = 9; VR = 4; NLP = 1

*AI, Artificial intelligence; R, Robotics; NLP, Natural language processing; CV, Computer vision; ML, Machine learning; HCI, Human-agent/computer/robot interaction; VR, Visualization and virtual reality; EEG, Electroencephalogram*.

These annotated observations were paired with data collected from facial, skeleton, or eye recognition tools (*n* = 9/20; 45%) (33, 37, 40–42, 44, 45, 50, 52), sensors (*n* = 6/20; 30%) (34, 40, 44, 45, 51, 53), EEG (*n* = 4/20; 20%) (34, 36, 39, 46), via distance estimates (*n* = 3/20; 15%) (47–49), and/or other tools (*n* = 6/20; 30%) such as microphones or electrodes (34, 38, 40, 43–45). To capture or predict participation, 18 of the 21 participation assessment approaches used multiple types of AI (86%) (34–50, 52). The vast majority of participation assessment approaches applied ML (*n* = 20/21; 95%) (34–53), followed by CV (*n* = 13/21; 62%) (33–35, 37, 41, 42, 44, 45, 47–50, 52), robotics and HCI (*n* = 9/21; 43%) (36, 37, 40, 43–45, 47–49), VR (*n* = 4/21; 19%) (34, 39, 46, 52) and NLP (*n* = 1/21; 5%) (38). ML and NLP were used to classify (i.e., automatically group based on prediction) data into categories of participation (e.g., engagement; non-engagement) (34–53). For example, Krupa et al. (51) paired physiological parameters (i.e., electro dermal activity and heart rate captured via sensors) with annotated observations of child participation to train a machine learning model to predict participation. CV was used to extract facial features (e.g., facial expressions, whether a face is directed toward the screen) to support capturing participation (33–35, 37, 41, 42, 44, 45, 47–50, 52). For instance, Kalantarian et al. (42) used a face tracker algorithm to locate the child's face within a video frame during a game session. These data [i.e., indicator for participation according to (42)] together with annotated observations on child participation were included in a ML model to predict child participation. VR, robotics and HCI were only used in combination with other types of AI (34, 36, 37, 39, 40, 43–49, 52). For example, Khamassi et al. (43) used ML to predict child participation by pairing data on a robot's expressivity with annotations on child participation. Similarly, Fan et al. (39, 46) used VR for driving simulations while participation was captured through paired annotated observations and collected EEG data entered in a predictive ML model.

In contrast to the pairing of annotated observation and recognition tools to collect data on child and youth participation, Ahmed et al. (33) captured participation using solely a threshold for facial action unit intensity, detected by a CV tool. This way of capturing participation was examined with children and youth with ASD completing self-contained academic lessons with tests (33).

Of the 21 included participation assessment approaches, 17 (81%) (34–37, 39, 40, 43–53) were delivered in person and outside the child or youth natural environment (e.g., laboratory) and 4 (19%) (33, 38, 41, 42) were either delivered remotely in the child's natural environment or the authors indicated their intention to deliver the developed assessment approach remotely. The purpose of assessing for child and/or youth participation in most studies was to contribute to building autonomous robots (*n* = 11/21; 52%) (36, 37, 40, 43–45, 47–50, 53) or systems that automatically adjust for task difficulty level based on the child and/or youth participation level (*n* = 4/21; 19%) (34, 39, 46, 52).

### Operationalizing Participation

We synthesize findings pertaining to how participation was operationalized per the terms used, and what was intended and actually captured.

None of the included assessment approaches used the term participation. Rather, most of them used the term engagement (*n* = 17/21; 81%) (33–46, 50, 52, 53), followed by social interaction (*n* = 3/21; 14%) (47–49) and involvement (*n* = 1/21; 5%) (51). About half of the included studies (*n* = 10/21; 48%) provided a definition for the term(s) used (33, 35–38, 43, 49, 50, 52, 53). Most assessment approaches examined participation in play activities (*n* = 8/21; 38%) (36, 38, 42, 47–50, 53), followed by driving (*n* = 3/21; 14%) (34, 39, 46), social interaction activities (*n* = 3/21; 14%) (40, 43, 52), academic lessons or mathematical problem solving (*n* = 2/21; 10%) (33, 35), and watching a movie (*n* = 1/21; 5%) (41). One study (5%) investigated assessing participation in varied activities (from simple games on smart phone to cycling) (51) and 3 studies (14%) (37, 44, 45) did not specify the activity.

In terms of how participation was intended to be captured, none of the included assessment approaches intended to capture attendance. To assess for involvement, only 1 participation assessment approach (5%) intended to capture both behavioral and emotional involvement (33), and only 1 assessment approach (5%) intended to capture emotional involvement (51) (see Table 3). The remaining 19 participation assessment approaches (91%) did not specify which aspects of involvement they intended to capture (34–50, 52, 53), so we assumed they intended to capture involvement generally.

**Table 3:** Operationalization of participation.

**References**		**Attendance**	**Involvement**			**Activity competence**	**Sense of Self**	**Preference**	**Environment/ context**	**Other**
			**Behavioral involvement**	**Cognitive involvement**	**Emotional involvement**					
Ahmed et al. (33)	Tried to measure		X		X					
	Actually measured		X							
Bian (34)	Tried to measure		X	X	X					
	Actually measured		X							X
Chorianopoulou et al. (38)	Tried to measure		X	X	X					
	Actually measured		X			X			X	X
Fan et al. (46)	Tried to measure		X	X	X					
	Actually measured		X							X
Fan et al. (39)	Tried to measure		X	X	X					
	Actually measured		X							X
Feil-Seifer et al. (47)	Tried to measure		X	X	X					
	Actually measured		X							
Feil-Seifer et al. (48)	Tried to measure		X	X	X					
	Actually measured		X							
Feil-Seifer et al. (49)	Tried to measure		X	X	X					
	Actually measured		X							
Feng et al. (40)	Tried to measure		X	X	X					
	Actually measured		X							
Fleury (36)	Tried to measure		X	X	X					
	Actually measured		X							X
Ge et al. (50)	Tried to measure		X	X	X					
	Actually measured		X							X
Hashemi et al. (41)	Tried to measure		X	X	X					
	Actually measured		X							
Kalantarian et al. (42)	Tried to measure		X	X	X					
	Actually measured		X							
Khamassi et al. (43)	Tried to measure		X	X	X					
	Actually measured		X						X	
Krupa et al. (51)	Tried to measure				X					
	Actually measured		X							X
Lahiri et al. (52)	Tried to measure		X	X	X					
	Actually measured		X							X
Liu et al. (53)	Tried to measure		X	X	X					
	Actually measured		X							X
Rudovic et al. (37)	Tried to measure		X	X	X					
	Actually measured		X							
Rudovic et al. (44)	Tried to measure		X	X	X					
	Actually measured		X			X				X
Rudovic et al. (45)	Tried to measure		X	X	X					
	Actually measured		X			X				X
Volta et al. (35)	Tried to measure		X	X	X					
	Actually measured		X							X
Total (*n*)	Tried to measure		20	19	21	0	0	0	0	0
	Actually measured		21	0	0	3	0	0	2	12

In terms of actual capturing of child and youth participation, all participation assessment approaches captured behavioral involvement. A total of 13 assessment approaches captured aspects not pertaining to participation (34–36, 38, 39, 43–46, 50–53) according to the used contemporary definition of participation (2, 3, 6–8). More specifically, 3 assessment approaches (14%) captured aspects that pertain to activity competences (i.e., quality of the doing such as quality of facial expression and actions on partner or object while playing or performing a non-specified activity) (38, 44, 45), 2 (10%) captured aspects of the environment and context (e.g., robot expressions) (38, 43), and 12 assessment approaches (57%) captured aspects that are outside of the scope of the fPRC framework (2, 3) and/or behavioral, cognitive, or emotional involvement (6–8) (e.g., heart rate, skin temperature) (34–36, 38, 39, 44–46, 50–53). None of the participation assessment approaches captured attendance, nor emotional or cognitive involvement.

## Discussion

Child and youth participation is a multidimensional and complex outcome in pediatric re/habilitation (2, 3, 9, 11, 18). Conceptually sound assessments are critical for shaping the enactment of the therapeutic processes (4, 5, 14, 18) and ensuring consistent interpretation of research findings across studies (5). While child and youth participation is characterized as both attendance and involvement (2, 3) (i.e., behavioral, cognitive, and emotional involvement) (6–8) few non-AI participation-focused assessments actually capture it as such (5). The increased use of AI in pediatric re/habilitation (17) provides an important opportunity for undertaking this scoping review, which examined how the concept of participation has been captured and operationalized in AI-based assessment approaches (4, 5, 14, 18) and identified gaps for future research at the intersection of pediatric re/habilitation and computer science, with potential for relevant extension into related fields [e.g., health informatics (15)].

### Lack of Reported Demographics and Sample Representativeness

Samples of included research were mainly skewed toward greater representation of male participants and children and youth with ASD and lacked reporting on family socio-economic status, family income, parental education, and child or youth race and ethnicity [Hispanic, non-Hispanic]. The concern of skewed data (e.g., oversampling of male participants and select diagnoses) as well as the lack of reporting on demographics in the training sets for applications of AI has been raised in prior literature (17, 54). Skewed and missing data in predictive models raise questions about their generalizability to the population and the degree they may reinforce existing inequalities in healthcare settings (54). Therefore, future research on this topic should consistently report on family socio-economic status and child and/or youth race and ethnicity as well as improve sampling strategies to better represent child or youth gender (55) and the range of diagnoses in children who experience unmet participation need (56, 57).

### Lack of AI-Based Participation Assessment Approaches Integrating the Child or Youth Perspective

All included AI-based assessment approaches integrated objective (i.e., observable) data to capture participation, with the vast majority using annotated observations. Only one of the 21 included participation assessment approaches integrated proxy-reported (e.g., caregiver-reported) data (40), and none of the included assessment approaches integrated child or youth self-reported data to capture participation. While the dominating focus on objective data is congruent with prior research not involving AI (4, 5, 58, 59), additional challenges for using AI to capture self-reported participation (15) may have amplified this result. Previously reported challenges include the lack of a machine-readable ontology describing components for activity and participation and the lack of annotation standards and data for this type of work (15). The dominating focus on objective data in this review might also be explained by the targeted population (i.e., mainly children and youth with ASD) that might be non-verbal communicators. Alternatively, it might be reflective of the common type(s) of AI employed (i.e., robotics, HCI, and VR) in participation-focused pediatric re/habilitation research (17). Many included participation assessment approaches employing robotics, HCI and VR aimed at integrating real-time participation data through using CV and/or ML (i.e. annotated observations) to simultaneously adapt the robotic behavior or VR task difficulty (34, 39, 43, 45, 47–49, 52).

The importance of the subjective dimension for participation assessment has been identified in prior research involving children and youth with acquired and congenital disabilities and their caregivers (5, 60–62). One way to include self-reported participation data in such applications might be through annotations conducted by children or youth themselves. However, due to interpersonal differences in experiencing and expressing participation (2, 3, 18, 44) generalization of such predictive models might be limited. Alternatively, non-AI participation assessments are often used within pediatric re/habilitation to gather self- or proxy-reported data for individual goal setting. For example, the Participation and Environment Measures (PEM) (63–65) assess for how often a child, youth or young adult participates in home, (pre-)school/daycare/work and community activities, their level of involvement in those activities, the desire for participation to change, applied participation-focused strategies, and the perceived impact of the environment on child, youth or young adult participation. Applications of AI such as recommender algorithms (e.g., constraint satisfaction and optimization) or NLP might provide simplified, more practical and low-cost ways for self- or proxy-reported data collection and interpretation for individual goal setting such as by systematically integrating responses into the individual child or youth participation profile, their participation goal, and intervention planning (12, 16, 66, 67).

### Lack of Remotely Administered AI-Based Assessment Approaches Capturing Participation

Few participation assessment approaches were administered remotely and in the natural environment [e.g., a child's home (38, 41)]. This result is surprising, due to the potential for leveraging technology to administer participation assessment approaches remotely and the previously reported importance of the environment in shaping child or youth participation (68–71). However, it aligns with results from a previous scoping review revealing only a few remotely delivered AI-based interventions targeting child or youth participation (17).

This result might be explained by the need for special equipment (e.g., camera equipped rooms) to administer the included AI-based participation-focused assessment approaches and interventions, with children and youth in attendance (17). Measuring participation with children and youth in attendance might also be the reason for the lack of measuring “attendance” in the included participation-focused assessment approaches. Alternatively, AI may be better suited to managing the higher complexity associated with assessing for a child's involvement when compared to their attendance (2, 3, 5). In pediatric re/habilitation, there are few non-AI participation-focused assessments that capture data on involvement which has been discussed as a key limitation (5). This review highlights early attempts to capture involvement through the use of AI, may reveal a unique opportunity for combining existing non-AI participation-focused assessments with new approaches using AI, to more fully assess child and youth participation. Therefore, there is need for further validation of these approaches to capturing involvement through AI (72).

### Lack of AI-Based Assessment Approaches Fully Aligned With Contemporary Definitions of Participation

While most included participation assessment approaches intended to capture all three aspects of involvement, none of them actually captured the two non-observable aspects of involvement (i.e., cognitive or emotional involvement). This mismatch between what was intended vs. actually captured might be connected to the lack of subjective data collection in the included participation assessment approaches as previously discussed. Subjective data could complement and/or extend recent efforts to quantify engagement, including behavioral engagement (58, 59).

**Figure 3:**
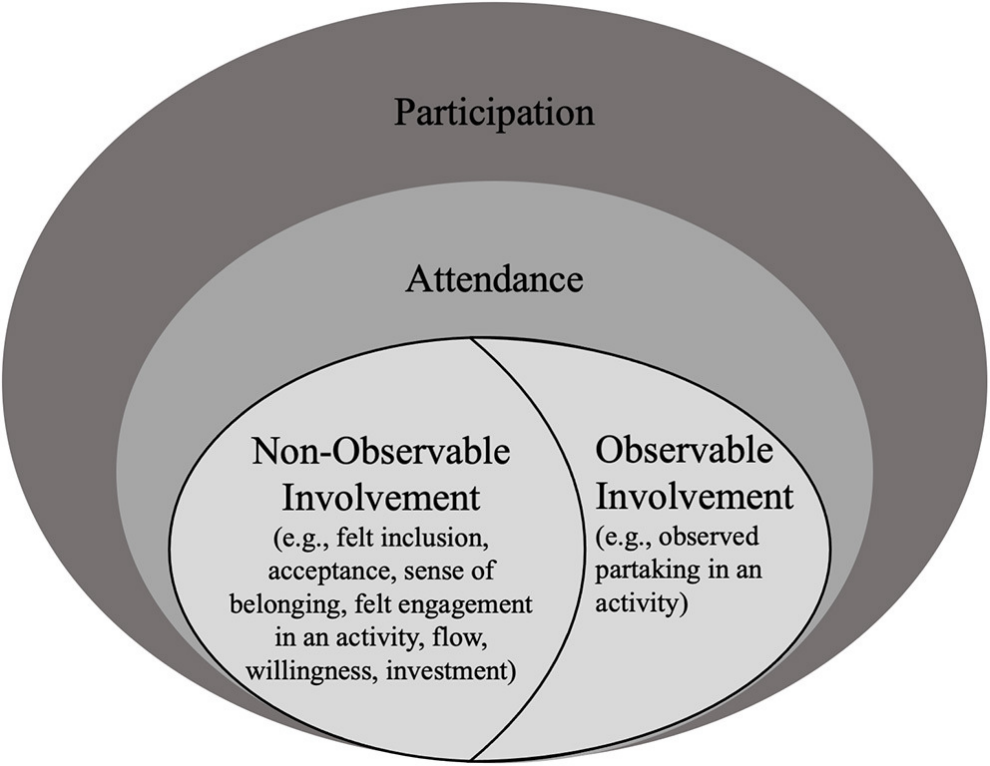
Participation encompassing observable and non-observable parts of involvement. Informed by the family of Participation-Related Constructs (fPRC) (2, 3) and research on the conceptualization of involvement (2, 3, 6–8, 72, 73).

Assessment approaches included in this scoping review captured aspects of activity competence and environment that were mistakenly labeled as participation or involvement. In addition, this review included a high number of participation assessment approaches that capture aspects that could not be mapped to the fPRC (2, 3). One such example are data on body functions such as kinematics data (50) that neither belong to the participation construct nor to participation-related constructs. One reason for researchers to collect body functions to capture participation might be related to the terms used to describe the types of involvement [i.e., behavioral, cognitive, emotional (6–8)]. In re/habilitation, the terms “behavioral, cognitive and emotional” have often been used in combination with “skills” such as concentration, attention, and asking questions (8), which arguably are body functions or activity competencies (15). Terms that have often been related to body functions or participation-related constructs (5) may misguide researchers and practitioners to focus on such constructs when designing assessments on involvement. Thus, findings of this review may indicate a need for different terms describing types of involvement. One way could be by differentiating between “observable” and “non-observable” aspects of involvement instead of using behavioral, cognitive, and emotional involvement as visualized in Figure 3. According to existing literature, observable involvement can be described as the observed partaking in an activity, for example, through focusing on a task. In contrast, non-observable involvement can be described as the feeling of inclusion, acceptance, and belonging as well as the felt engagement in an activity such as through flow or investment (5, 8, 73, 74).

The distinction between observable and non-observable aspects of involvement may also help to emphasize the importance to include subjective data to fully capture participation, which has been identified as a limitation in existing participation assessments with and without the use of AI (5). Because knowledge on the construct involvement is still emerging (73), future research on child and youth involvement in attended activities is needed for its conceptualization in participation assessments in pediatric re/habilitation.

### Limitations

The main limitation of this research is the risk of having missed relevant documents. For example, when AI was not mentioned in the title or abstract, that document was likely excluded from our search and/or when applying our selection criteria. Additionally, we did not evaluate the quality of included studies. However, this is not typically done in scoping reviews due to their purpose of providing a map of existing evidence vs. synthesizing the best available evidence (21).

## Conclusions

There is an increasing number of research studies on the use of AI to capture participation involvement, which indicates the promise of AI to capture participation and an opportunity to further investigate the construct of participation, particularly child and youth involvement. Our results show that most of the included assessment approaches captured participation through observation and by applying ML, CV or robotics and HCI. There was a mismatch between what assessment approaches intended to capture and what they actually captured, with a high number of assessments collecting data unrelated to participation, according to contemporary frameworks of child and youth participation (2, 3, 6–8). Our results suggest 4 main gaps that need to be addressed in future research: (1) a lack of research reporting on common demographic factors and including samples representing the population of children and youth with a congenital or acquired disability; (2) lack of AI-based participation assessments integrating the child or youth perspective; (3) lack of remotely administered AI-based assessments capturing both the attendance and involvement dimensions of child and youth participation; and (4) lack of AI-based assessments that fully align with contemporary definitions of child and youth participation.

## Data Availability Statement

The original contributions presented in the study are included in the article/Supplementary Material, further inquiries can be directed to the corresponding author/s.

## Author Contributions

VK mentored by her committee members (MK, NP, DA, and AB), took the lead in conceptualizing the study, and drafting all sections of the manuscript. MV and VK screened articles based on their title and abstract. ZS, JS, and VK screened articles based on full-text reads and extracted data from included articles and VK synthesized them to gain the results of this research. DA and AB provided feedback on the conceptualization of the study and prior versions of this manuscript. MK and NP co-mentored VK through each step of this work including study conceptualization, systematic literature search, study selection process, data analysis, and write-up of this manuscript. All authors provided editing of the manuscript and read and approved the final version.

## Funding

This work was conducted in partial fulfillment of the requirements for a Ph.D. in Rehabilitation Sciences and was supported by the University of Illinois at Chicago, through their Dean's Scholar Fellowship (PI: VK) and Chancellor's Undergraduate Research Award (ZS). The contents of this manuscript were developed under a grant from the National Institute on Disability, Independent Living, and Rehabilitation Research (NIDILRR grant number 90SFGE0032-01-00). NIDILRR is a Center within the Administration for Community Living (ACL), Department of Health and Human Services (HHS). The contents of this manuscript do not necessarily represent the policy of NIDILRR, ACL, or HHS, and you should not assume endorsement by the Federal Government.

## Conflict of Interest

The authors declare that the research was conducted in the absence of any commercial or financial relationships that could be construed as a potential conflict of interest.

## Publisher's Note

All claims expressed in this article are solely those of the authors and do not necessarily represent those of their affiliated organizations, or those of the publisher, the editors and the reviewers. Any product that may be evaluated in this article, or claim that may be made by its manufacturer, is not guaranteed or endorsed by the publisher.

## Abbreviations

AI: artificial intelligence
ASD: autism spectrum disorder
EEG: electroencephalogram
fPRC: family of participation-related constructs
HCI: human-agent/computer/robot interaction
ML: machine learning
NLP: natural language processing
PEM: participation and environment measure
VR: virtual reality.
