# Supplementary material for: Capturing and Operationalizing Participation in Pediatric Re/Habilitation Research Using Artificial Intelligence: A Scoping Review
Source: Front Rehabil Sci. 2022 Apr 14;3:855240. doi: 10.3389/fresc.2022.855240 (PMC9340801; doi:10.3389/fresc.2022.855240)
Supplement: Supplementary file 1 [file Data_Sheet_1.docx]

Supplementary Material

Appendix 1. Exemplar Search History for Grey Literature Searches.

| Database | Search terms |
| --- | --- |
| ProQuest Dissertation & Theses | (MAINSUBJECT.EXACT("artificial intelligence") OR MAINSUBJECT.EXACT("data mining") OR MAINSUBJECT.EXACT("algorithms") OR MAINSUBJECT.EXACT("fuzzy logic") OR AB,TI("artificial intelligence") OR AB,TI("machine learning") OR AB,TI("natural language processing") OR AB,TI("data mining") OR AB,TI("algorithm*") OR AB,TI("fuzzy logic") OR AB,TI("knowledge bases") OR AB,TI("neural networks") OR AB,TI("deep learning") OR AB,TI("affective computing") OR AB,TI("cognitive computing") OR AB,TI("knowledge engineering") OR AB,TI("knowledge representation") OR AB,TI("semantic networks") OR AB,TI("reinforcement learning") OR AB,TI("inductive logic programming") OR AB,TI("unsupervised learning") OR AB,TI("supervised learning") OR AB,TI("semi-supervised learning") OR AB,TI("pattern recognition") OR AB,TI("feature extraction") OR AB,TI("image analysis") OR AB,TI("text analysis") OR AB,TI("expert systems") OR AB,TI("robot*") OR AB,TI("knowbot*") OR AB,TI("data processing") OR AB,TI("predictive model") OR AB,TI("virtual agent") OR AB,TI("chatbot") OR AB,TI("virtual reality") OR AB,TI("computer vision") OR AB,TI("constraint satisfaction") OR AB,TI("constraint optimization") OR AB,TI("game theory") OR AB,TI("human computation") OR AB,TI("knowledge-based agent") OR (AB,TI("classifier") AND (AB,TI("logistic regression") OR AB,TI("Naïve Bayes") OR AB,TI("Decision trees")))) AND (MAINSUBJECT.EXACT("pediatrics") OR MAINSUBJECT.EXACT("elementary school student") OR MAINSUBJECT.EXACT("Kindergarten students") OR AB,TI("child*") OR AB,TI("adolesc*") OR AB,TI("youth*") OR AB,TI("student*") OR AB,TI("teen*") OR AB,TI("young adult") OR AB,TI("young adults") OR AB,TI("paediatric*") OR AB,TI("pediatric*") OR AB,TI("toddler*") OR AB,TI("infant*") OR MAINSUBJECT.EXACT("caregivers") OR MAINSUBJECT.EXACT("family") OR MAINSUBJECT.EXACT("parenting") OR AB,TI("caregiv*") OR AB,TI("parent*") OR AB,TI("family") OR AB,TI("families")) AND (MAINSUBJECT.EXACT("Disorders") OR AB,TI("disab*") OR AB,TI("special needs") OR AB,TI("special need") OR AB,TI("handicap*") OR AB,TI("impair*") OR AB,TI("disorder*")) AND (MAINSUBJECT.EXACT("participation") OR MAINSUBJECT.EXACT("mainstreaming (Education)") OR AB,TI("participation") OR AB,TI("engag*") OR AB,TI("attendance") OR AB,TI("involvement") OR AB,TI("inclus*")) |
|  | (MAINSUBJECT.EXACT("artificial intelligence") OR MAINSUBJECT.EXACT("data mining") OR MAINSUBJECT.EXACT("algorithms") OR MAINSUBJECT.EXACT("fuzzy logic") OR AB,TI("artificial intelligence") OR AB,TI("machine learning") OR AB,TI("natural language processing") OR AB,TI("data mining") OR AB,TI("algorithm*") OR AB,TI("fuzzy logic") OR AB,TI("knowledge bases") OR AB,TI("neural networks") OR AB,TI("deep learning") OR AB,TI("affective computing") OR AB,TI("cognitive computing") OR AB,TI("knowledge engineering") OR AB,TI("knowledge representation") OR AB,TI("semantic networks") OR AB,TI("reinforcement learning") OR AB,TI("inductive logic programming") OR AB,TI("unsupervised learning") OR AB,TI("supervised learning") OR AB,TI("semi-supervised learning") OR AB,TI("pattern recognition") OR AB,TI("feature extraction") OR AB,TI("image analysis") OR AB,TI("text analysis") OR AB,TI("expert systems") OR AB,TI("robot*") OR AB,TI("knowbot*") OR AB,TI("data processing") OR AB,TI("predictive model") OR AB,TI("virtual agent") OR AB,TI("chatbot") OR AB,TI("virtual reality") OR AB,TI("computer vision") OR AB,TI("constraint satisfaction") OR AB,TI("constraint optimization") OR AB,TI("game theory") OR AB,TI("human computation") OR AB,TI("knowledge-based agent") OR (AB,TI("classifier") AND (AB,TI("logistic regression") OR AB,TI("Naïve Bayes") OR AB,TI("Decision trees")))) AND (MAINSUBJECT.EXACT("pediatrics") OR MAINSUBJECT.EXACT("elementary school student") OR MAINSUBJECT.EXACT("Kindergarten students") OR AB,TI("child*") OR AB,TI("adolesc*") OR AB,TI("youth*") OR AB,TI("student*") OR AB,TI("teen*") OR AB,TI("young adult") OR AB,TI("young adults") OR AB,TI("paediatric*") OR AB,TI("pediatric*") OR AB,TI("toddler*") OR AB,TI("infant*")) AND (MAINSUBJECT.EXACT("participation") OR MAINSUBJECT.EXACT("mainstreaming (Education)") OR AB,TI("participation") OR AB,TI("engag*") OR AB,TI("attendance") OR AB,TI("involvement") OR AB,TI("inclus*")) AND (MAINSUBJECT.EXACT("Rehabilitation") OR AB,TI("healthcare") OR AB,TI("health care")) |
